# Salivary Calculus

**Published:** 1860-07

**Authors:** H. B. Burnham

**Affiliations:** Epping, New Hampshire.


### ARTICLE XXV.

*Salivary Calculus.* By H. B. BURNHAM, M. D., Epping, New Hampshire.

J. H., aged 48 years, of spare habit and slender constitution, some fourteen years since was seized with a severe pain under the left side of his tongue. He applied to his family physician, who could give him no satisfactory information as to the cause or nature of his complaint: neither could he afford him any relief. He was induced to consult other physicians in his vicinity, and he did so with like results. In the meantime, a small tumor made its appearance on the under side of his tongue, near or at the seat of pain.

He went to Boston and consulted the late Dr.———, who informed him that his disease was cancer, and gave him but little encouragement as to any permanent relief. He returned to his home, determined to abide the result of what he then supposed an incurable disease. From that time until about the first of February last, he has suffered paroxysms of severe and excruciating pain at different times.

The tumor gradually increased in size, and the paroxysms of pain became more frequent, until it finally became inflamed, suppurated and burst, discharging a small quantity of pus and a calculus weighing fifteen grains, having the general appearances of ordinary renal or biliary calculi. He has since been entirely free from pain.

[*Boston Med. and Surg. Journal.*

